# Supplementary material for: Quantitative prediction of variant effects on alternative splicing in MAPT using endogenous pre-messenger RNA structure probing
Source: eLife. 2022 Jun 13;11:e73888. doi: 10.7554/eLife.73888 (PMC9236610; doi:10.7554/eLife.73888)
Supplement: Supplementary file 1. [file elife-73888-supp1.docx]

**Supplementary file 1**

**ANOVA of Exon 10 PSI for between individuals versus within an individual**

|  | SS | df | MS | F | Pr(>F) |
| --- | --- | --- | --- | --- | --- |
| Between individuals | 11.76 | 145 | 0.08108 | 1.944 | 2.31e-09 |
| Within individuals | 50.64 | 1214 | 0.04171 |  |  |
