# Supplementary material for: Quantitative prediction of variant effects on alternative splicing in MAPT using endogenous pre-messenger RNA structure probing
Source: eLife. 2022 Jun 13;11:e73888. doi: 10.7554/eLife.73888 (PMC9236610; doi:10.7554/eLife.73888)
Supplement: Supplementary file 7. [file elife-73888-supp7.docx]

**Supplementary file 7**


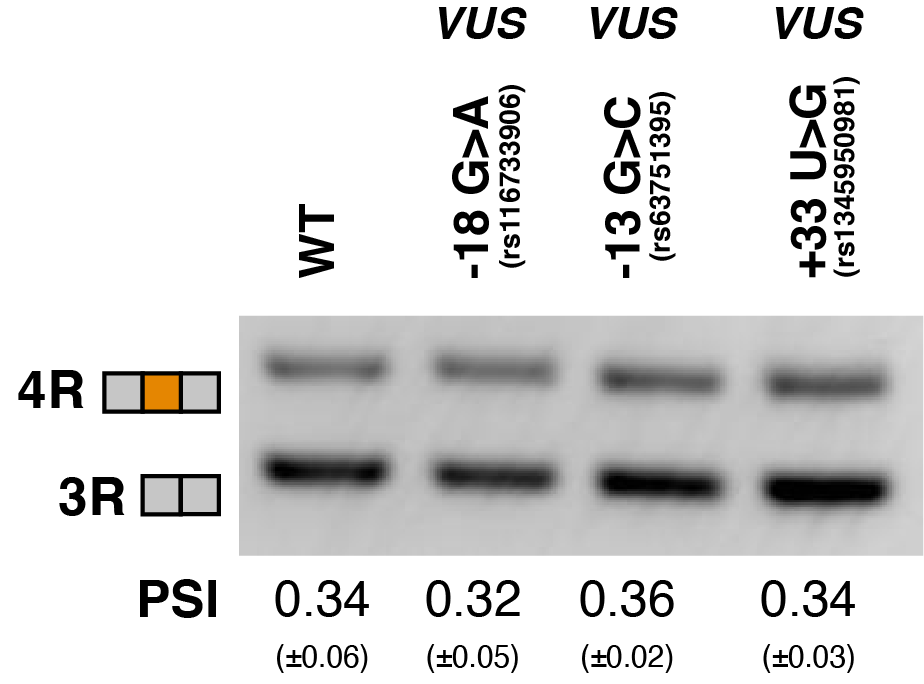


Representative gel of RT-PCR data for splicing assay in the presence of WT VUSs. Splicing reporter was transfected into HEK293 cells. The mean Exon 10 PSI displayed for each variant was calculated from three replicates and standard error is shown in brackets below.
