# Supplementary material for: Quantitative prediction of variant effects on alternative splicing in MAPT using endogenous pre-messenger RNA structure probing
Source: eLife. 2022 Jun 13;11:e73888. doi: 10.7554/eLife.73888 (PMC9236610; doi:10.7554/eLife.73888)
Supplement: Source data 1. [file elife-73888-data1.pdf]

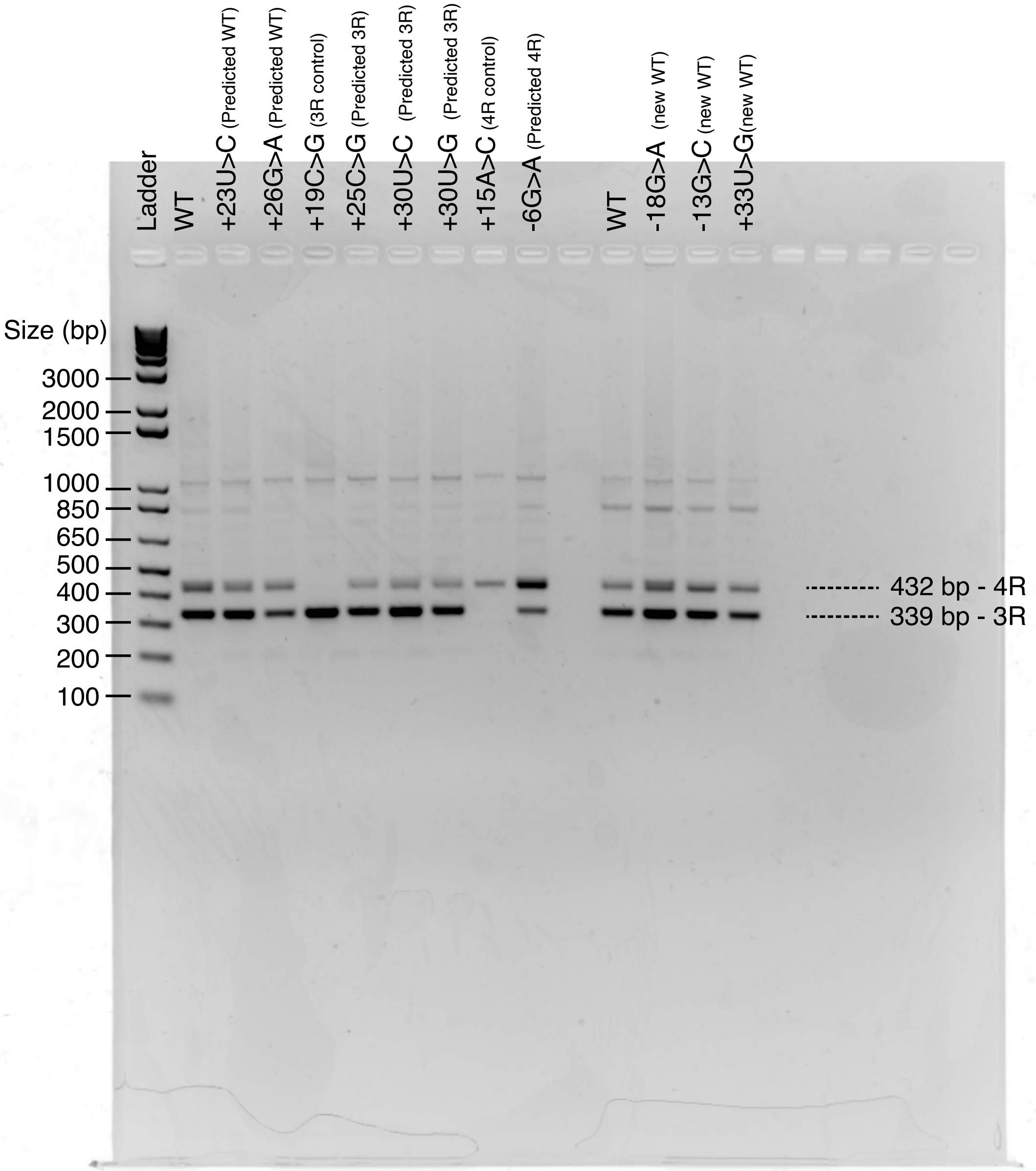

Rep1 - MAPT Splicing Assay
2% agarose gel in 1X tris-acetate EDTA (TAE) buffer

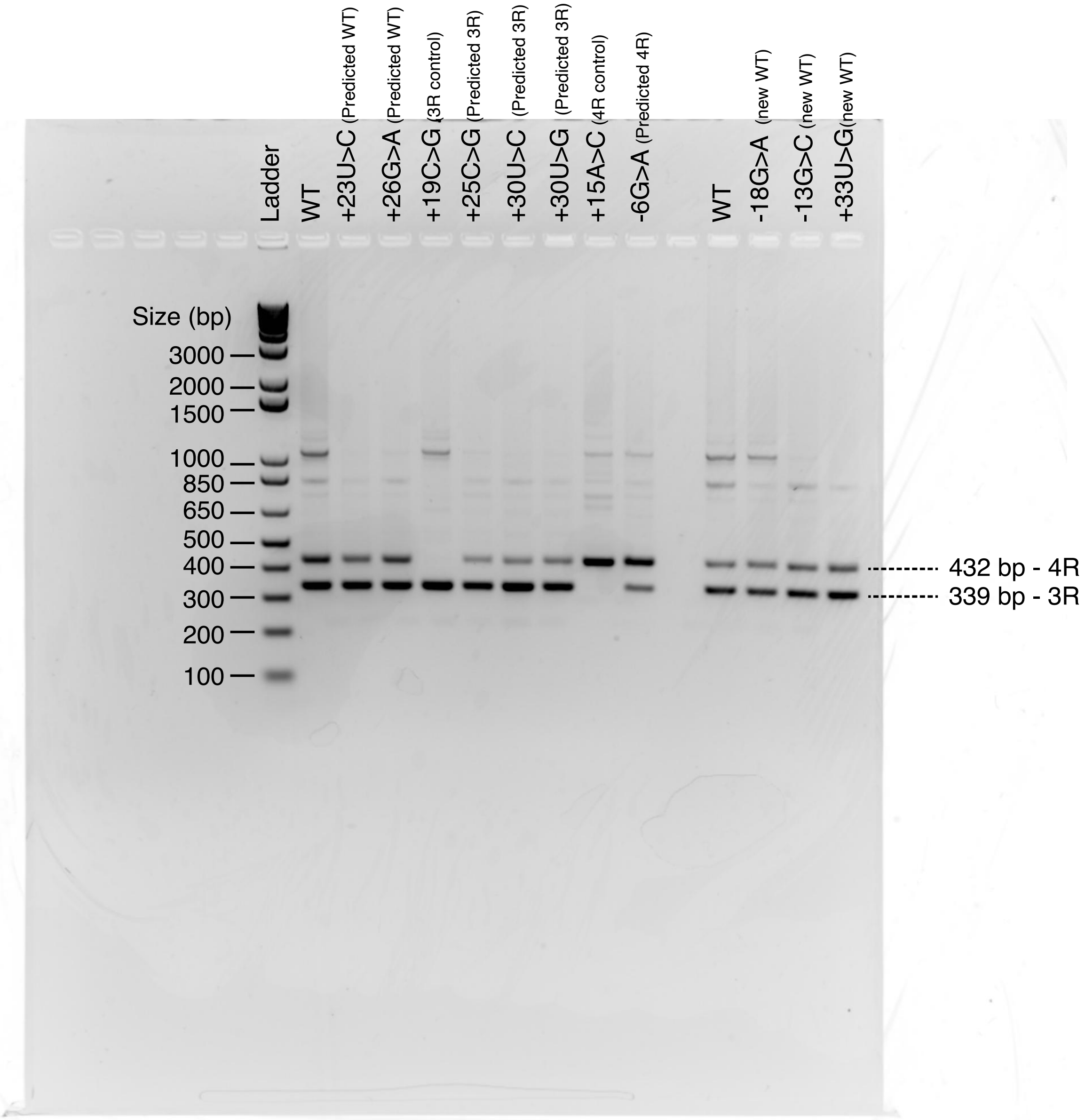

Rep 2 - MAPT Splicing Assay
2% agarose gel in 1X tris-acetate EDTA (TAE) buffer

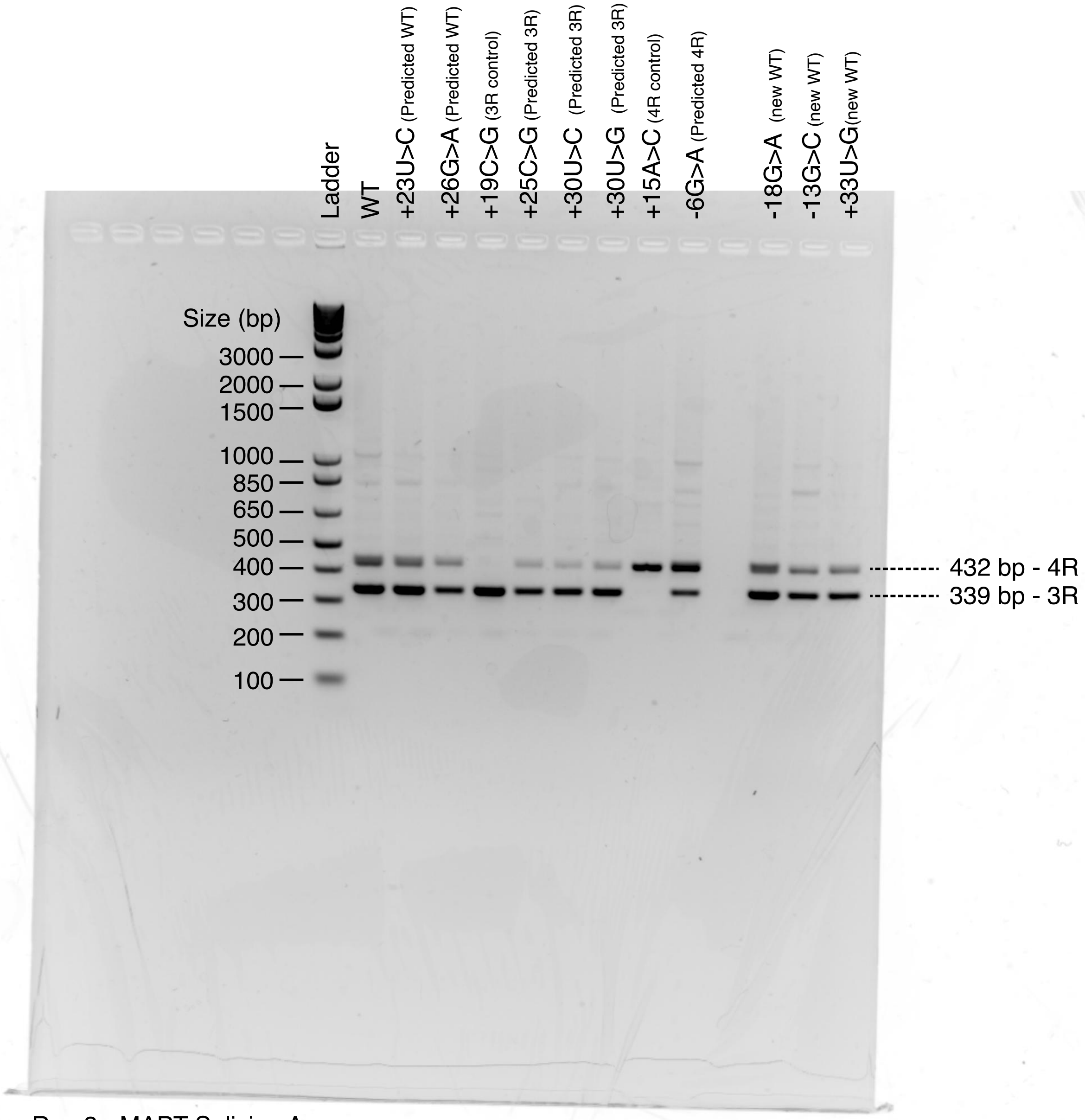

Rep 3 - MAPT Splicing Assay
2% agarose gel in 1X tris-acetate EDTA (TAE) buffer
