# Supplementary figures and images for: Quantitative prediction of variant effects on alternative splicing in MAPT using endogenous pre-messenger RNA structure probing
Source: eLife. 2022 Jun 13;11:e73888. doi: 10.7554/eLife.73888 (PMC9236610; doi:10.7554/eLife.73888)

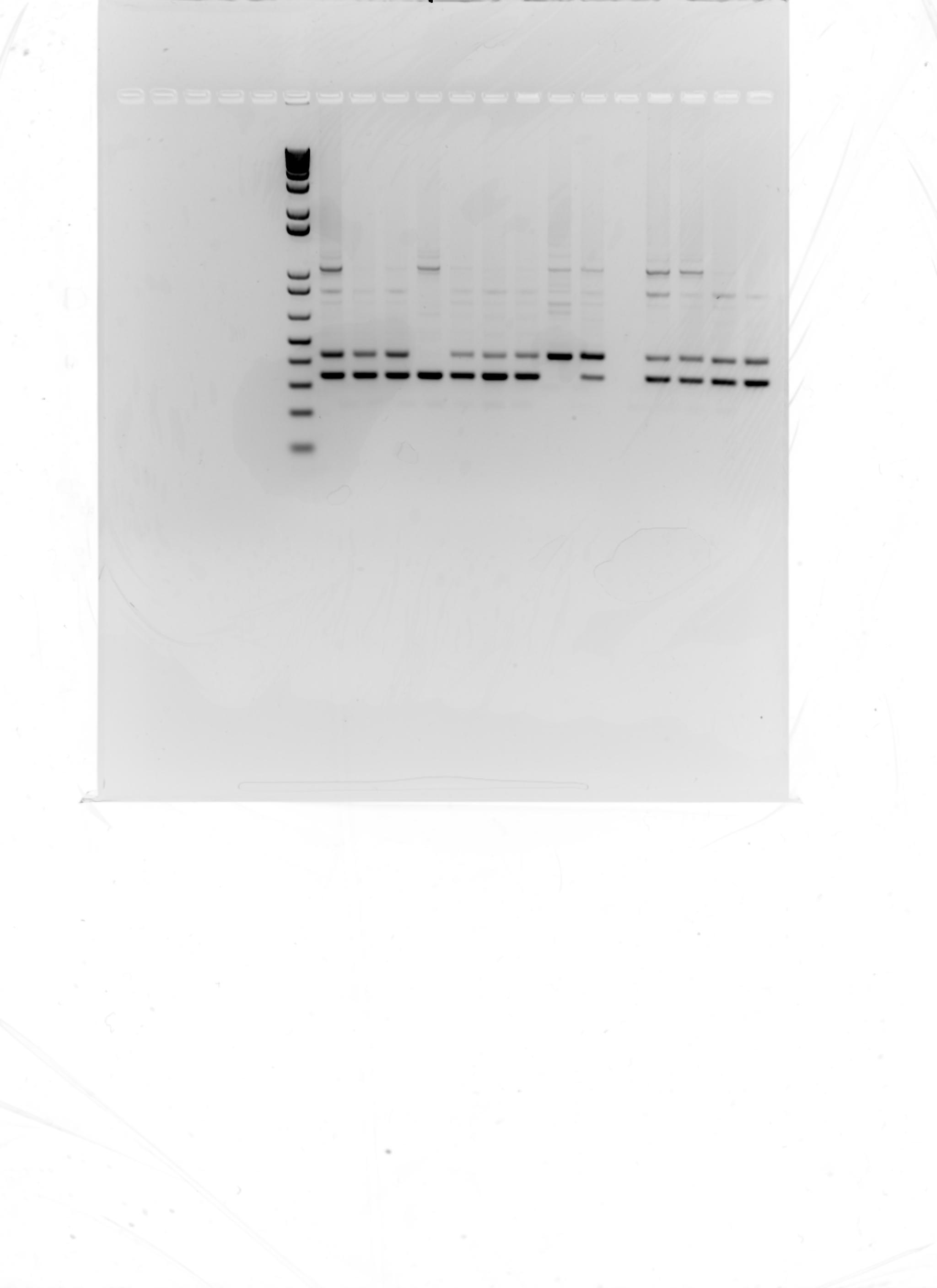

Supplement: Source data 2. [file elife-73888-data2.zip › Gel_Images_Raw/MAPT_splicingAssay_Variants_Rep2_2021.09.07_12.23.47_Fl-UV.jpg]

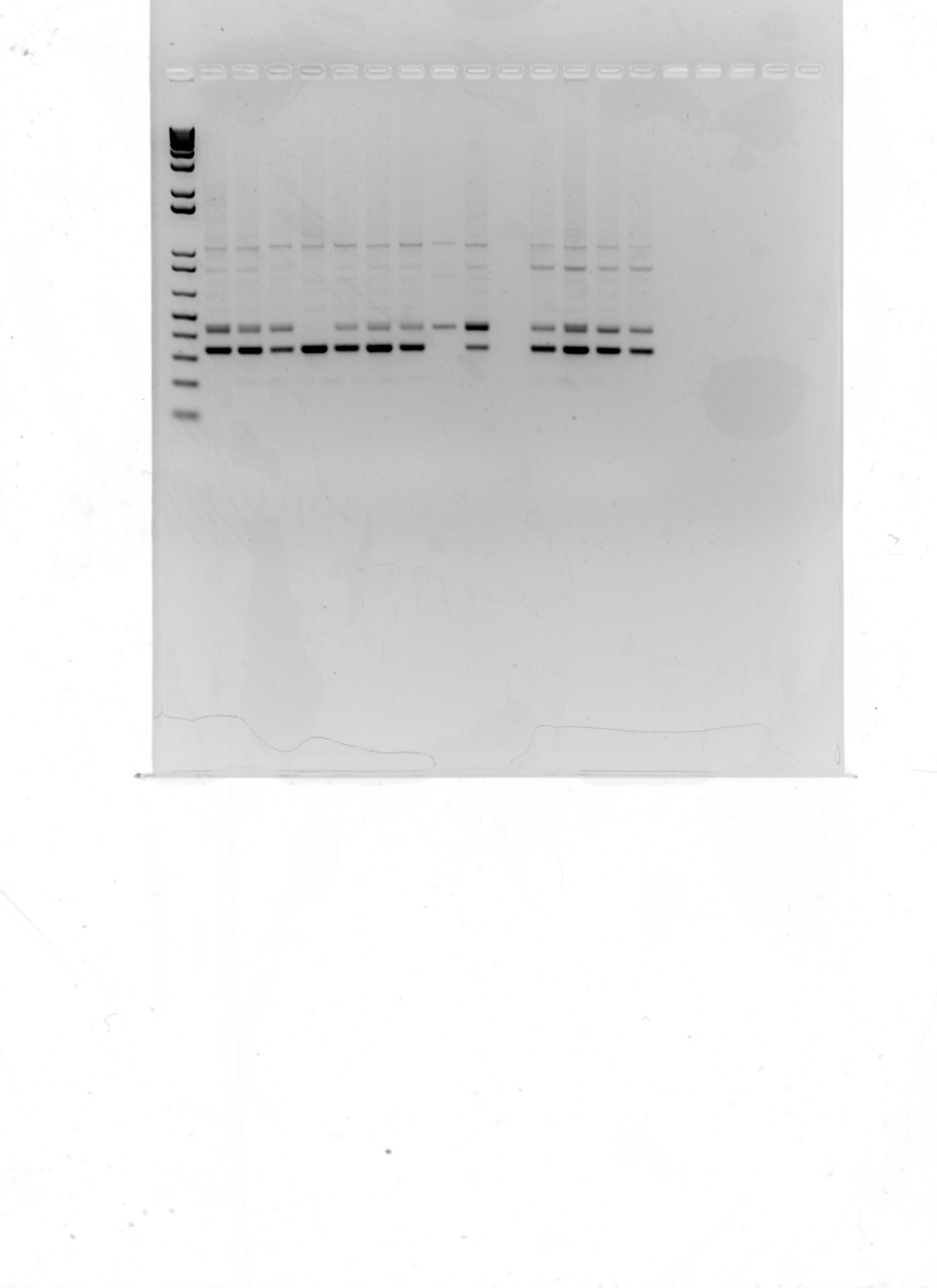

Supplement: Source data 2. [file elife-73888-data2.zip › Gel_Images_Raw/MAPT_splicingAssay_Variants_Rep1_ 2021.09.07_15.53.47_Fl-UV.jpg]

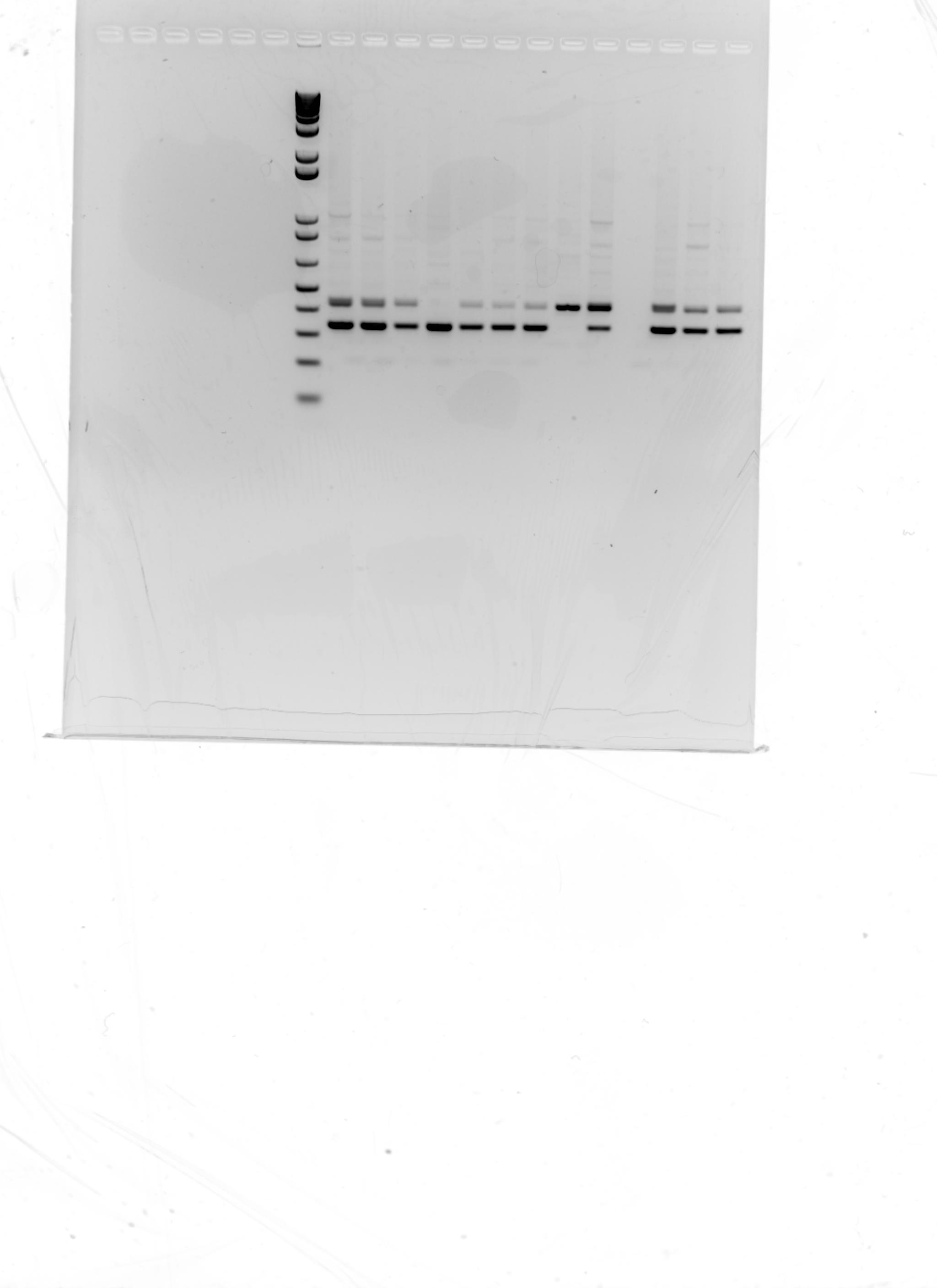

Supplement: Source data 2. [file elife-73888-data2.zip › Gel_Images_Raw/MAPT_splicingAssay_Variants_Rep3_ 2021.09.08_11.24.19_Fl-UV.jpg]
