# Supplementary material for: Quantitative prediction of variant effects on alternative splicing in MAPT using endogenous pre-messenger RNA structure probing
Source: eLife. 2022 Jun 13;11:e73888. doi: 10.7554/eLife.73888 (PMC9236610; doi:10.7554/eLife.73888)
Supplement: Transparent reporting form [file elife-73888-transrepform1.pdf]

Westbrook Centre
Milton Road
Cambridge CB4 1YG, UK
P 01223 855340
W elifesciences.org
T @elife

eLife's transparent reporting form

We encourage authors to provide detailed information *within their submission* to facilitate the interpretation and replication of experiments. Authors can upload supporting documentation to indicate the use of appropriate reporting guidelines for health-related research (see EQUATOR Network), life science research (see the BioSharing Information Resource), or the ARRIVE guidelines for reporting work involving animal research. Where applicable, authors should refer to any relevant reporting standards documents in this form.

If you have any questions, please consult our Journal Policies and/or contact us: editorial@elifesciences.org.

Sample-size estimation

- You should state whether an appropriate sample size was computed when the study was being designed
- You should state the statistical method of sample size computation and any required assumptions
- If no explicit power analysis was used, you should describe how you decided what sample (replicate) size (number) to use

Please outline where this information can be found within the submission (e.g., sections or figure legends), or explain why this information doesn't apply to your submission:

For the analysis of Exon 10 PSIs across tissues and individuals, PSIs were only calculated if there were at least 10 reads across the MAPT exon10-intron 10 junction. This is in line with previous published analysis of exon-intron junctions (Song, Botvinnik et al., 2017). For ANOVA analysis, only individuals with data in more than 7 tissues were considered. This information can be found in Materials and Methods.
This does not apply for DMS-MaP structure probing datasets as data is averaged across millions of cells in line with previously published DMS-MaP methodology (Homan et al., 2014)

Replicates

- You should report how often each experiment was performed
- You should include a definition of biological versus technical replication
- The data obtained should be provided and sufficient information should be provided to indicate the number of independent biological and/or technical replicates
- If you encountered any outliers, you should describe how these were handled
- Criteria for exclusion/inclusion of data should be clearly stated
- High-throughput sequence data should be uploaded before submission, with a private link for reviewers provided (these are available from both GEO and ArrayExpress)

Please outline where this information can be found within the submission (e.g., sections or figure legends), or explain why this information doesn't apply to your submission:

Information about replicates can be found in Figure 1 – figure supplement 2 and Figure 2 – figure supplement 1 and their associated legends.
Information about the exclusion of DMS reactivity data can be found in Materials and Methods under the section “Updating DMS parameters for RNAstructure using SSU ribosome data from T47D cells”.
High-throughput sequencing data has been uploaded to SRA and can be viewed at https://dataview.ncbi.nlm.nih.gov/object/PRJNA762079?reviewer=ccd0ed8huijlgqhd625453vr6f

eLife Sciences Publications, Ltd is a limited liability non-profit non-stock corporation incorporated in the State of Delaware, USA, with company number 5030732, and is registered in the UK with company number FC030576 and branch number BR015634 at the address Westbrook Centre, Milton Road, Cambridge CB4 1YG.

Statistical reporting

- Statistical analysis methods should be described and justified
- Raw data should be presented in figures whenever informative to do so (typically when N per group is less than 10)
- For each experiment, you should identify the statistical tests used, exact values of N, definitions of center, methods of multiple test correction, and dispersion and precision measures (e.g., mean, median, SD, SEM, confidence intervals; and, for the major substantive results, a measure of effect size (e.g., Pearson's r, Cohen's d)
- Report exact p-values wherever possible alongside the summary statistics and 95% confidence intervals. These should be reported for all key questions and not only when the p-value is less than 0.05.

Please outline where this information can be found within the submission (e.g., sections or figure legends), or explain why this information doesn't apply to your submission:

> Beta Regression models are described in Materials and methods and the regression summary output for the different models can be found in Supplementary file 6.
> Raw data for splicing assay gels can be found in Figure 6 – figure supplement 1F.
> Pearson's R^2 has been calculated for all Experimental Vs Predicted comparisons of Exon 10 PSI and can be found in Figures 3B, 4B,C and 5A as well as in Supplementary file 6.
>
> Wilcoxon Rank sum test was used to compare bootstrapped R^2 values and details can be found in Figure 3C and its associated legend. It was additionally used to compare predicted PSIs of all variants around 100nt window of exon10-intron10 junction and known disease variants: this can be found in Figure 6B and its associated legend.
>
> Fisher exact test was used to compare proportions in PSI drivers between non-disease variants and disease variants and information about this can be found in Figure 6C and its associated legend.

(For large datasets, or papers with a very large number of statistical tests, you may upload a single table file with tests, Ns, etc., with reference to sections in the manuscript.)

Group allocation

- Indicate how samples were allocated into experimental groups (in the case of clinical studies, please specify allocation to treatment method); if randomization was used, please also state if restricted randomization was applied
- Indicate if masking was used during group allocation, data collection and/or data analysis

Please outline where this information can be found within the submission (e.g., sections or figure legends), or explain why this information doesn't apply to your submission:

> For comparing disease vs non-disease variants, we allocated a variant in the disease group if the variant was found in the Human Gene Mutation Database v2016.

Additional data files ("source data")

- We encourage you to upload relevant additional data files, such as numerical data that are represented as a graph in a figure, or as a summary table
- Where provided, these should be in the most useful format, and they can be uploaded as "Source data" files linked to a main figure or table
- Include model definition files including the full list of parameters used

eLife Sciences Publications, Ltd is a limited liability non-profit non-stock corporation incorporated in the State of Delaware, USA, with company number 5030732, and is registered in the UK with company number FC030576 and branch number BR015634 at the address Westbrook Centre, Milton Road, Cambridge CB4 1YG.

- Include code used for data analysis (e.g., R, MatLab)
- Avoid stating that data files are "available upon request"

Please indicate the figures or tables for which source data files have been provided:

Source data files have been provided for Figures 1A,1B,1C, 2A,2B, 4A, 5C, 6A, 6D.
Feature data can be found in supplementary file 6.
Information about previously validated *MAPT* mutations and dbSNP variants can be found in supplementary file 2 and 3 respectively.
Code for feature generation and regression analysis is available at https://git.io/JuSW8

eLife Sciences Publications, Ltd is a limited liability non-profit non-stock corporation incorporated in the State of Delaware, USA, with company number 5030732, and is registered in the UK with company number FC030576 and branch number BR015634 at the address Westbrook Centre, Milton Road, Cambridge CB4 1YG.
